# Performance of generative artificial intelligence models in the theoretical exam for the Brazilian Board-Certified Dermatology title (TED): a comparative study^☆^

**DOI:** 10.1016/j.abd.2026.501326

**Published:** 2026-03-25

**Authors:** Matheus Alves Pacheco, Athos Paulo Santos Martini

**Affiliations:** Hospital Universitário, Universidade Federal de Santa Catarina, Florianópolis, SC, Brazil

Dear Editor,

Artificial Intelligence (AI)-based language models, such as ChatGPT, have been widely explored in medicine, demonstrating performance comparable to that of physicians and students in national and international exams (USMLE, PLAB, HKMLE, NMLE, and Revalida). Meta-analyses also indicate that some of these systems have statistically lower average accuracy than specialists, but comparable to that of non-specialist physicians.^1^, ^2^, ^3^, ^4^

In dermatology, AI models have been evaluated in diverse contexts, assessing their diagnostic accuracy against published clinical cases, in North American specialty exams, and in the Dermatology Board-certified Specialist Titles (TED, *Título de Especialista em Dermatologia)* applied by the Brazilian Society of Dermatology (SBD, *Sociedade Brasileira de Dermatologia*).^5^, ^6^, ^7^

Despite the growing scientific production in the area, systematic comparisons between different generative AI platforms are still scarce. Thus, the present study aims to evaluate and compare the performance of multiple generative AI tools in solving the TED 2025 theoretical exam, aiming to contribute to the discussion about their potential role as a complementary instrument in specialized medical training and dermatological clinical reasoning.

An observational, cross-sectional, and retrospective study with a quantitative approach was conducted to evaluate the performance of different generative AI models on the TED 2025 theoretical exam, which consisted of 80 multiple-choice questions with four alternatives (A, B, C, D) and only one correct answer, covering various topics in dermatology. Eight generative AI models were included, with their characteristics being described in Table 1.

**Table 1:** Characteristics of the generative AI models used in the study.

AI model	Development Company	Country	Free?* (data from July 2025)	Image Upload?
GPT-3.5	OpenAI	United States	Yes	No
Grok 3	xAI	United States	Yes, limited	Yes
Meta Llama 4	Meta	United States	Yes	Yes
Gemini 2.5 Flash	Google	United States	Yes	Yes
Claude Sonnet 4	Anthropic	United States	Yes	Yes
Le Chat Mistral AI	Mistral AI	France	Yes, limited	Yes
DeepSeek V3	DeepSeek AI	China	Yes	Yes
GPT 4.5	OpenAI	United States	No	Yes

The initial prompt was the same for all models: “You are taking the Dermatology Board-certified Specialist Title (TED) exam – first phase. This is a multiple-choice question with four alternatives (A, B, C, or D). Read the question carefully and select only the correct alternative. Do not explain the reason for your answer. Your answer must contain only one capital letter: A, B, C, or D.”

The interactions were conducted in July 2025. For each question, the complete statement – ​​including text, alternatives, and, when applicable, supplementary images (in models that supported image uploads) – was provided to the AI. Only one question required image interpretation. No additional information was provided beyond the original content of the exam. The generated answers were recorded and compared with the official answer key released by the examining board. Accuracy was calculated as the proportion of correct answers in relation to the total number of questions.

The analyses were performed on the Julius AI platform, using Cochran's Q test for overall comparison and McNemar's test with Holm adjustment for pairwise comparisons.

Claude Sonnet 4 led with 72/80 correct predictions, followed by Gemini 2.5 Flash (71/80) and GPT-4.5 (69/80). DeepSeek V3, Meta LLaMA 4, GPT-3.5, and Grok 3 obtained between 63–64 correct predictions. Le Chat Mistral AI had the lowest performance (59/80; Fig. 1).

**Fig. 1:**
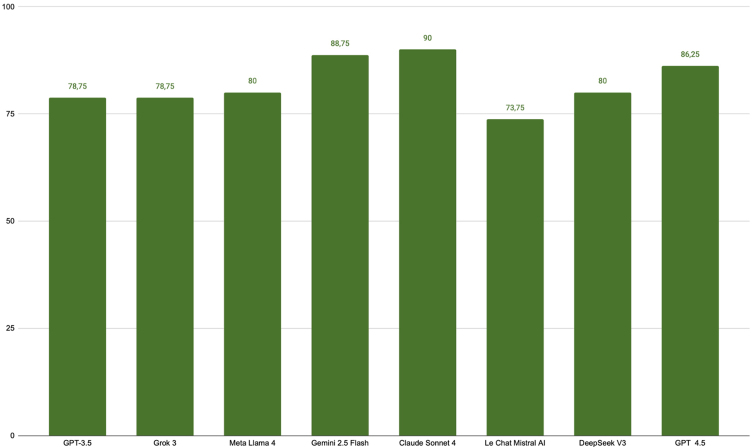
Percentage of correct answers for the eight generative AI models evaluated in the TED 2025 theoretical exam.

The difference between the most accurate and least accurate models was 16.25 percentage points, highlighting heterogeneity in performance between platforms. Despite this variation, all models showed an accuracy rate above 70%, indicating a resolution capacity consistently above the level expected by chance.

Cochran's Q test indicated a statistically significant difference between the eight models assessed (p = 0.0119). However, paired comparisons using McNemar's test, with Holm's adjustment for multiple comparisons, did not identify significant differences between specific pairs of models. These results suggest heterogeneity in overall performance, but without statistically robust evidence of consistent superiority of one model over another.

The present study expands on the findings of Jabour et al. (2024), who evaluated only ChatGPT-3.5.^6^ By including multiple generative AI models, the authors demonstrate that these are capable of high performance in solving TED 2025 questions, with accuracy rates exceeding 70% across all tested models. Notably, Claude Sonnet 4 (90%) and Gemini 2.5 Flash (88.75%) showed accuracy comparable to that of experts, highlighting the potential of these tools.

It is worth noting, however, that the performance of the models was not homogeneous. The difference of up to 16.25 percentage points between the highest and lowest performing models reinforces the need for independent validation before future clinical incorporation. Such heterogeneity was also described in a study that observed significant variations in the performance of different AIs in medical examinations conducted in distinct cultural contexts.^2^

It was observed that 37 of the 80 questions showed no variability in responses, being answered identically by all models. This pattern is possibly related to the difficulty profile of the TED 2025, with 38.8% of the items considered easy or very easy and more than 60% showing good or very good discrimination capacity, according to the official SBD report.^8^ Although this balance is desirable for evaluating human candidates, in the present study, it may have reduced the sensitivity of statistical tests to detect point differences between AI models, since easy-to-solve questions tend to generate uniform responses.

Similar results have already been described in the literature. In Brazil, a study reported that Chat GPT-4.0 outperformed students and non-specialist physicians in solving questions from the national progress exam, with an accuracy rate of 87.2%.^1^ In a study conducted in the United States, it was demonstrated that AI models outperformed resident physicians in official medical certification exams.^5^ Specifically in dermatology, a study showed that Chat GPT-4.0 achieved performance similar to that of medical students when solving simulated questions from US board exams.^3^

In Brazil, Jabour et al. (2024) demonstrated that Chat GPT-3.5 was able to solve 69% of the questions on the TED 2023.^6^ Pacheco and Martini (2025) reinforced that AI performance is even more relevant when complemented by clinical and histopathological data, a context in which the models showed accuracy greater than 80% when evaluating real published clinical cases.^7^

Therefore, generative models are capable of applying clinical reasoning, even in the face of highly complex examinations for specialists. These findings reinforce the promising potential of generative AIs. Future studies should evaluate their performance in real care contexts and integrate multimodal data and ethical and regulatory debate, contributing to the basis for the discussion on their incorporation into practice.

## ORCID ID

Matheus Alves Pacheco: 0000-0003-3427-3536

## Financial support

This research has not received any specific funding from public, private, or non-profit funding agencies.

## Authors' contributions

Athos Paulo Santos Martini: Design and planning of the study; drafting and editing of the manuscript or critical review of important intellectual content.

Matheus Alves Pacheco: Drafting and editing of the manuscript or critical review of important intellectual content.

## Research data availability

The entire dataset supporting the results of this study was published in this article.

## Conflicts of interest

None declared.
